# What proportion of *Salmonella* Typhi cases are detected by blood culture? A systematic literature review

**DOI:** 10.1186/s12941-016-0147-z

**Published:** 2016-05-17

**Authors:** Vittal Mogasale, Enusa Ramani, Vijayalaxmi V. Mogasale, JuYeon Park

**Affiliations:** Policy and Economic Research Department, Development and Delivery Unit, International Vaccine Institute, Seoul, Republic of Korea; Epidemiology Department, Development and Delivery Unit, International Vaccine Institute, Seoul, Republic of Korea; Biostatistics and Data Management Department, Development and Delivery Unit, International Vaccine Institute, Seoul, Republic of Korea

## Abstract

Blood culture is often used in definitive diagnosis of typhoid fever while, bone marrow culture has a greater sensitivity and considered reference standard. The sensitivity of blood culture measured against bone marrow culture results in measurement bias because both tests are not fully sensitive. Here we propose a combination of the two cultures as a reference to define true positive *S.* Typhi cases. Based on a systematic literature review, we identified ten papers that had performed blood and bone marrow culture for *S*. Typhi in same subjects. We estimated the weighted mean of proportion of cases detected by culture measured against true *S.* Typhi positive cases using a random effects model. Of 529 true positive *S.* Typhi cases, 61 % (95 % CI 52–70 %) and 96 % (95 % CI 93–99 %) were detected by blood and bone marrow cultures respectively. Blood culture sensitivity was 66 % (95 % CI 56–75 %) when compared with bone marrow culture results. The use of blood culture sensitivity as a proxy measure to estimate the proportion of typhoid fever cases detected by blood culture is likely to be an underestimate. As blood culture sensitivity is used as a correction factor in estimating typhoid disease burden, epidemiologists and policy makers should account for the underestimation.

## Background

Typhoid fever is a fecal oral transmitted systemic disease caused by the *Salmonellaenterica* serovar Typhi. Culturing the bacteria from body fluids is the definitive test for the diagnosis of typhoid fever although inconclusive serological methods such as Widal test are commonly employed in many health care settings [1, 2]. The culture of *S.* Typhi can be done from many body fluids such as blood, bone marrow, urine, rose spot extracts, duodenal aspirates and stool, while the blood culture remains the mainstay of definitive diagnosis [1, 3]. However, blood culture sometimes does not identify the bacteria even if it exists in the blood because of many procedural and technical issues. It is known that the maximum blood culture yield will be achieved when bacteremia is at peak such as in the first to third week from the onset of the illness [1] The quantity of blood sample collected may play a role, with a higher quantity more likely to give bacterial growth. Specimen collection, storage and transportation condition is likely to affect blood culture yield besides the culture media used [1, 4, 5]. Furthermore, febrile patients often take antibiotics; self-administered, prescribed or un-prescribed, likely to reduce the possibility of bacterial growth because antibiotics may inhibit the growth of *S.* Typhi. Considering the sub-optimal diagnostic yield of blood culture, studies that estimate typhoid fever disease burden tend to apply “a blood culture sensitivity correction factor” to account for missed cases [6, 7].

The sensitivity of a test is defined as the probability that the test correctly classifies people with disease as positive [8]. In patients with typhoid fever, the blood culture sensitivity measures the proportion of the *S*. Typhi cases detected by blood culture compared to an independent test, commonly bone marrow culture. A previous literature review on the sensitivity of blood culture suggested 40 to 60 % [9] acknowledging the widely accepted sensitivity rate of 50 % [1] which is often used as a correction factor in many disease burden studies [6, 7, 10, 11]. The blood culture sensitivity is measured against bone marrow culture which is often considered superior because it can yield bacteria among those who took antibiotics and even after the bacteremia subsides in the blood [1, 12]. Ironically, bone marrow culture is also subject to same methodological and technical limitations as blood culture and misses some typhoid fever cases besides posing clinical difficulties as an invasive procedure. There are a few studies that document typhoid fever cases that are bone marrow negative and blood culture positive [13–15]. One of the clinical reviews has suggested a sensitivity of 40–80 % for blood culture and 55–67 % for bone marrow culture [3]. This indicates that the current practice of using blood culture sensitivity as a proxy to represent proportion of cases detected in disease burden studies has intrinsic bias of using a sub-optimal reference test against which it is measured.

Besides being used as a correction factor in the measurement of disease burden estimates which play crucial role in policy making, the proportion of typhoid fever cases detected is important for clinicians. Even if the blood culture is negative for typhoid fever, the person still may be suffering from the disease and need treatment. Thus understanding how many typhoid fever cases are actually detected by blood culture has critical importance for clinicians who would treat the patients, epidemiologists who estimate the disease incidence and policy makers who would use the data for decisions on control measures. As we argued that the blood culture sensitivity measured against bone marrow culture is not the true measure of the proportion of typhoid fever cases detected, here we present a new method. This method will be helpful in measuring the precise proportion of typhoid fever cases detected when blood culture is deployed as a diagnostic test.

## Methods

First, we chose two tests, blood culture and bone marrow culture to define an algorithm to estimate the proportion of bacteremic typhoid fever cases identified by blood culture. As noted before, both tests do not identify all typhoid fever cases [3] and hence choosing one of them as a reference standard to compare against the other would be an imperfect estimation of the proportion of cases detected. Therefore, we considered a composite reference standard of two tests to define true positive cases based on epidemiological principles [16, 17] as described below.

Anybody who tested positive for typhoid fever either in blood or bone marrow culture was considered a true positive and the proportion of cases identified by each test was measured against true positive cases (Eq. 1). That means we could use only those subjects in whom both blood culture and bone marrow cultures were performed. This method allows the measurement of the proportion of cases detected by each test in comparison to true positive (Eqs. 2, 3). We compared our results to the sensitivity of blood culture compared against bone marrow culture results (Eq. 4). The decision matrix is shown in Table 1. The calculation can be represented as below.

**Table 1:** General decision matrix for blood and bone marrow diagnostic test

		BMC^a^	
		+ve	−ve
BC^a^	+ve	TP_BC, BMC_	FP_BC_ OR FN_BMC_
	−ve	FN_BC_ OR FP_BMC_	TN_BC, BMC_

*BC* blood culture, *BMC* bone marrow culture, *TP* true positive, *FP* false positive, *FN* false negative, *TN* true negative

^a^Biological materials withdrawn from same patient

1 $$ProCase_{i} = \frac{{TP_{i} + FP_{i} }}{{FN_{i} + TP_{j\& i} + FN_{j} }}\,$$

2 $$ProCase_{BC} = \frac{{TP_{BC} + FP_{BC} }}{{FN_{BC} + TP_{BMC\& BC} + FN_{BMC} }}$$

3 $$ProCase_{BMC} = \frac{{TP_{BMC} + FP_{BMC} }}{{FN_{BC} + TP_{BMC\& BC} + FN_{BMC} }}$$

4 $$ProCase_{BC vs. BMC} = \frac{{TP_{BC} + FP_{BC} }}{{TP_{BMC} + FP_{BMC} }}$$

where,

*ProCase*_*i*_ = Proportion of typhoid fever cases detected by culture technique i in comparison to true positive cases detected based on both tests i and j.

*ProCase*_*BC vs BMC* =_ Proportion of typhoid fever cases detected by blood culture in comparison to typhoid fever cases detected by bone marrow culture.

*TP*_*i*_ = True positive cases by culture technique i (culture test shows bacterial growth by culture technique i when the bacteria is present in the sample based on culture technique j).

*FP*_*i*_ = False positive cases by culture technique i (culture test shows bacterial growth by culture technique i when no bacteria was detected in the sample by culture technique j).

*FN*_*i*_ = False negative cases by culture technique i (culture test shows no bacteria growth in the sample by culture technique i when bacteria is present in the sample based on culture technique j).

*TP*_*j*&*i*_ = True positive test for both culture techniques j and i (culture test shows bacterial growth in culture technique j when the bacteria is present in the sample based on culture technique i).

*FN*_*j*_ = False negative cases by culture technique j (culture test shows no bacteria growth in the sample by culture technique j when bacteria is present in the sample based on culture technique i).

*BC* = Blood culture test.

*BMC* = Bone marrow culture test.

A systematic review of literature was conducted to assess the proportion of typhoid fever cases identified by blood culture. The search involved three databases; Medline as a primary electronic database, followed by Embase, WHO and Pan American Health Organisation (PAHO) websites to identify additional publications. The search was limited to studies published in English language, published before December 31, 2013 among human subjects. The key words used were (“typhoid” OR “typhoid fever” OR “*Salmonella* Typhi” OR “*S*. Typhi” OR “*Salmonella* infection” OR “enteric fever”) AND (“blood” OR “blood culture” OR “culture of blood” OR “diagnostics” OR “sensitivity” OR “positivity”). The inclusion and exclusion criteria are given in Table 2. The search was conducted by an independent researcher; the results were verified by a second researcher for inclusion and exclusion criteria matching. Any differences between two researchers were resolved based on discussion and agreement, if unresolved, third independent researcher made the final decision. All selected papers were reviewed by a third researcher before data extraction to confirm its adherence to inclusion criteria. In the final list, we included papers that conducted both blood and bone marrow culture in same set of patients.

**Table 2:** Inclusion and exclusion criteria

Inclusion criteria
Listed in PubMed, Embase database, WHO or PAHO databases
Published before 31st December 2013
Conducted in human subjects
Published in English language
Collected blood and bone marrow samples from same patients for *S*. Typhi detection
Identified by search terms defined in the text
Study design: Laboratory surveillance
Exclusion criteria
Papers that do not distinguish *S*. Typhi from *S*. Paratyphi in blood culture results

Last, the selected publications were reviewed to calculate true positive, false positive, true negative and false negative cases from blood and bone marrow culture results in the same patients. We estimated the case weighted mean proportion of *S*. Typhi detected from selected studies using random effects model. The observations on culture media, volume of blood sample collected and duration of illness were presented descriptively.

## Results

**Fig. 1:**
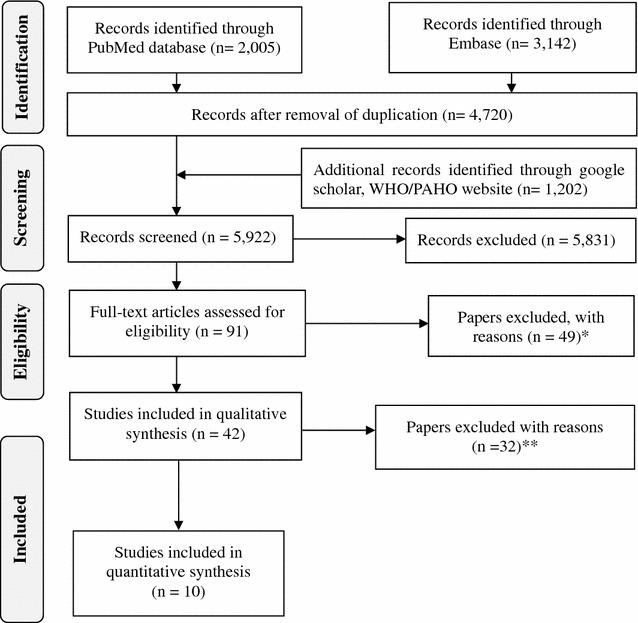
PRISMA diagram for systematic literature review. *Asterisk* No conformity to inclusion criteria because 35 papers used single diagnostic test, either blood or bone marrow; 7 were review papers, 1 used animal sample for investigating the impact of* S*. Typhimurium on human and 6 papers were inaccessible. *Double Asterisk* 29 had used only blood culture for typhoid fever confirmation; three papers did not differentiate results for *S.* Typhi and *S.* Paratyphi A

The systematic literature search in PubMed and Embase as well as WHO and PAHO websites produced 5922 papers. A total 5831 studies were excluded because of duplication and not fitting with inclusion criteria on review of the title and abstract (Fig. 1). Of the remaining 91 papers, six full-text articles were inaccessible, 64 papers used a single diagnostic test (either blood or bone marrow), seven were review papers, one used animal samples for investigating the impact of *S.* Typhimurium on humans, and three papers did not differentiate results for *S.* Typhi and *S.* Paratyphi A. Finally, 10 papers were selected including one from Western Africa [13], one from South Africa [18], one from South Asia [19], four from South-East Asia [14, 20–22] and three from Latin America [15, 23, 24] (Table 3).

**Table 3:** *S*. Typhi isolation using blood and bone marrow cultures as sensitivity standard from 10 locations

Location	Study period	No. of subjects included	No. of subjects tested by both BC and BMC	*S*. Typhi detected by BC or BMC	TP_BC_ (a)	FP_BC_ (b)	FN_BC_ (c)	TN_BC_ (d)	TP_BMC_ (e)	FP_BMC_ (f)	FN_BMC_ (g)	TN_BMC_ (h)	Total *S*. Typhi recovery from BC [x] (a + b)	Total *S*. Typhi recovery from BMC [y] (e + f)	Culture media used	Specimen volume
Zaria, Nigeria [13]	1986–1987	64	31	22	8	3	11	9	8	11	3	9	11	19	B and BM: thioglycolate broth	B: 2 ml; BM: 1–2 ml
Transvaal, S. Africa [18]	<1951	40	28	18	13	0	5	10	13	5	0	10	13	18	Oxbile and nutrient broths	NP
Karachi, Pakistan [19]	<1990	100	100	88	58	0	30	12	58	30	0	12	58	88	B and BM: thioglycolate/BHIB	B: 5 ml; BM: 0.5–1 ml
Semarang, Indonesia [14]	1989–1990	145	80	80	39	13	28	0	39	28	13	0	52	67	B and BM: oxgall broth	B: 3 and 10 ml; BM:1 ml
Semarang, Indonesia [20]	<2001	61	61	54	43	1	10	7	43	10	1	7	44	53	Luria-Betani (LB) broth	B: 8–10 ml; BM: 1–2 ml
Jakarta, Indonesia [21]	<1992	52	52	37	17	5	15	15	17	15	5	15	22	32	B: oxgall	B: 3 ml; BM; 0.5–0.8 ml
Dong Thap, Vietnam [22]	1993–1999	110	103	73	53	4	16	30	53	16	4	30	57	69	B: oxgall, oxbile, BHIB	B: 5 and 15 ml; BM: 1 ml
Mexico City, Mexico [23]	<1975	68	62	57	24	1	32	5	24	32	1	5	25	56	B: peptone broth; BM: Peptone/Ruiz-castenada	B: 2 ml; BM: NP
Lima, Peru [24]	<1979	66	60	57	26	0	31	3	26	31	0	3	26	57	B&BM: Trypticase-soy broth/Ruiz-Castaneda	B: 5 ml; BM: 1 ml
Lima, Peru [15]	1984	118	58	43	9	10	24	0	9	24	10	0	19	36	B&BM: oxgall	B: 3 ml; BM: 0.5 ml
Total	–	824	635	529	290	37	202	91	290	202	37	91	327	495	–	–

*BC* blood culture, *BMC* bone marrow culture; *TPBC* True positive blood culture cases; *FPBC* False positive blood culture cases, *FNBC* False negative blood culture, *TPBMC* True positive bone marrow culture, *FNBMC* False negative bone marrow cultures, *NP* Not presented, *B* blood, *BM* bone marrow, *BHIB* brain–heart-infusion broth

**Fig. 2:**
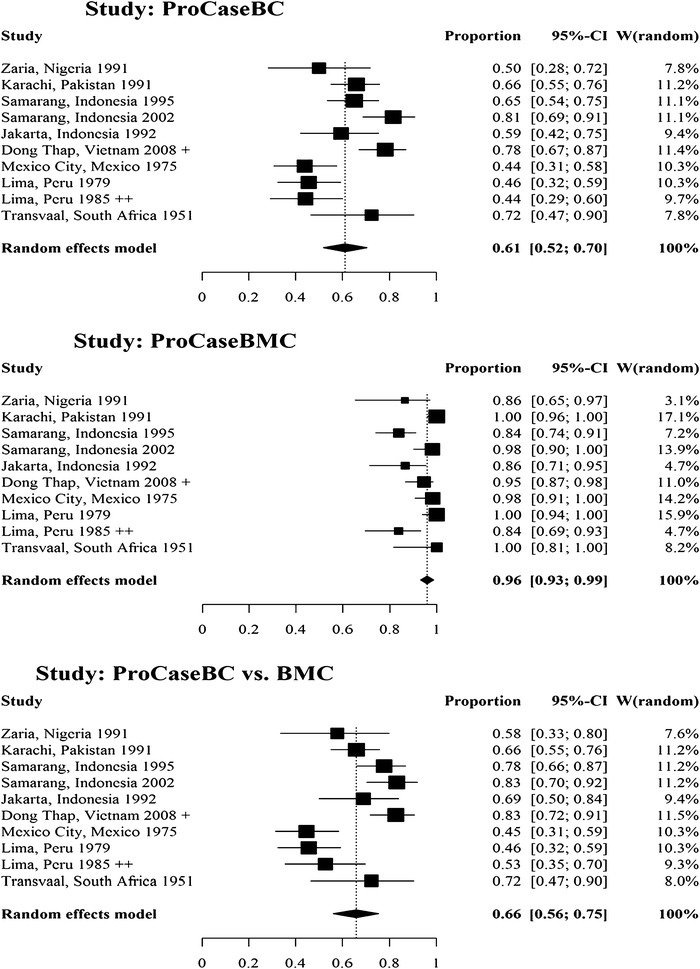
*Forest plot* for the proportion of *S*. Typhi detected and for blood culture sensitivity based on ten studies identified in the systematic literature review. *ProcaseBC* Proportion of* S*. Typhi positive blood cultures among true positive* S*. Typhi positive cases (either blood or bone marrow cultures positive for* S*. Typhi when specimens were collected from same patients), *ProCaseBMC* Proportion of S. Typhi positive bone marrow cultures among true positive* S*. Typhi positive cases (either blood or bone marrow cultures positive for* S*. Typhi when specimens were collected from same patients; *ProCaseBC vs. BMC* Sensitivity of blood culture measured against bone marrow culture positive cases as reference standard

The ten studies tested 635 people for *S*. Typhi using both blood and bone marrow cultures. Of 529 true *S*. Typhi positive cases the proportion of *S.* Typhi detection was 61 % (95 % CI 52–70 %) and 96 % (95 % CI 93–99 %) for blood and bone marrow culture respectively (Fig. 2). The sensitivity of blood culture was found to be 66 % (95 % CI 56–75 %) when bone marrow culture results were used as the reference standard comparator.

The recovery of *S*. Typhi varied depending upon the bacteriological culture media used [21, 22], the volume of blood samples collected [22] and duration of the disease [15, 24]. The Luria-Betani (LB) broth media resulted in better recovery compared to Difco Oxgal with Selenite F broth media (44/54 vs. 19/43 respectively) [15, 20]. Nevertheless the lower yield from Difco Oxgal broth could be because many studies used blood volumes of less than 3 ml and the same media showed improved recovery where ≥5 ml blood was used [14, 22]. Some studies showed that the collection of a 15 ml of blood sample increased the sensitivity of blood culture comparable to that of the bone marrow culture [9, 22]. The earlier a patient reports to a health facility, the better the recovery of *S*. Typhi organism in blood cultures and bone marrow cultures [14, 20, 22].

## Discussion

This review presents a summary of the proportion of *S.* Typhi cases detected by blood culture based on the combined results of blood and bone marrow culture in the same individuals. The results indicate that 2 out of 5 people infected with *S.* Typhi would remain undiagnosed if blood culture were deployed as the diagnostic test. The results also imply that even if bone marrow culture were used, 1 in 25 people infected with *S.* Typhi would remain undiagnosed. Clinicians should be aware and remember these missed diagnoses in case management. It is likely that even the combination of blood culture and bone marrow will not diagnose all patients with typhoid fever but in the absence of a perfect reference standard test this is difficult to confirm. Most importantly, the findings indicate that the proportion of typhoid fever cases detected by blood culture is lower than the blood culture sensitivity measured against bone marrow culture. This has important public health ramifications in typhoid fever control because blood culture sensitivity is often used as s a proxy for the proportion of *S.* Typhi detected in correcting disease burden estimates.

Previous global disease burden estimates [10, 11] and incidence studies [6, 7] have used 50 % blood culture sensitivity as a proxy measure for the proportion of cases detected by blood culture while this review suggests a higher value of 61 %. If this higher proportion were used as a correction factor in estimating the incidence, the disease burden decreases. In recent typhoid disease burden estimations in low and middle income countries, the authors have clearly demonstrated these differences through a sensitivity analysis. The study estimated 25.3 million cases when 50 % blood culture sensitivity correction was used which declined to 20.6 million cases when 61 % blood culture correction was deployed [25]. Such variation in the disease burden estimation may influence global policy and financing decisions, hence careful considerations in using these results are necessary. Notably we argue that the blood culture sensitivity should not be used as a proxy for the proportion of *S*. Typhi cases detected because it undermines the disease burden estimates. In this review we found blood culture sensitivity was 66 % using bone marrow culture as the reference standard. If we use this sensitivity in previous disease burden estimates [25], the typhoid fever burden in low and middle income countries decreases by 1.5 million; from estimated 20.6 million to 19.1 million.

Our study has several limitations which should be considered carefully before applying the summary information to clinical, epidemiological and policy decisions. First, it is well known that the bacterial growth in culture is dependent on the time from the onset of illness and sample collection; greater recovery was observed in early bacteremic phase. We could not account for time factor in our analysis as such detailed information was unavailable in the papers identified. It is possible that some studies could have collected samples early after the disease onset and others in a later stage resulting in inter observational biases. Had we known the time of sample collection for all studies, we could have presented sensitivity as a factor of time. Second, sampling the same febrile case in our analysis may not mean that blood and bone marrow samples were collected simultaneously. We only know that same person had provided both blood and bone marrow culture samples. As commonly practiced, bone marrow sample might have been collected in a later stage of the disease management or after antibiotic prescription. In this case, it could be possible that those who were tested negative for blood culture might have been sampled for bone marrow which would create a blood culture sensitivity underestimation bias. Third, the volume of specimen collected will have differed between blood and bone marrow and between different studies which might have introduced the bias in bacterial isolation as larger the volume of the sample, higher the yield would be. Studies of the quantitative bacteriology of the blood and bone marrow suggest that the bacterial counts in bone marrow may be 10-fold higher than blood so larger volumes of blood maybe comparable with smaller volumes of bone marrow aspirate [26]. Many studies identified in this review were conducted several decades ago and had collected smaller volume of blood as per the prevalent practices at that time. Similarly, different culture media were employed in different studies which may have influenced the results as the potential for bacterial growth may differ between the culture media. None of the studies identified by this review used the commercial media that are now commonly used for culture [27]. Also, as these studies were conducted in different time periods (1955–2001), in different geographical regions, in different endemicity locations, and in different clinical and laboratory settings; inter study variation may have influenced the results. Forth, three papers did not separate reports for *S*. Typhi and *S*. Paratyphi A cases, which led to the exclusion of those papers from the analysis. We cannot predict if the inclusion of such studies would have altered the results. Fifth, as both blood and bone marrow cultures are partially sensitive, the composite reference standard may have still missed some typhoid fever cases. In that situation, the proportion or the *S.* Typhi detected could be an overestimation. The true proportion of cases detected and true sensitivity of culture can only be measured against a perfect test that is fully sensitive and specific. Molecular diagnostic tests may hold the promise for future to provide better composite reference standard, if not perfect [3]. Sixth, as bone marrow culture is an invasive procedure, there is an intrinsic procedural difficulty that may have discouraged the clinicians from performing the procedure; or may have restrained patients or guardians from giving procedural consent. This may have introduced differential sample collection bias between blood and bone marrow culture. We could not measure this bias in our study because information on failed procedures or people did not consent for procedure was not presented in the papers. Seventh, the blood and bone marrow cultures could only be performed in health facilities sufficiently equipped for the complexity of both procedures and with accessible skilled personnel. This means the selected papers could represent the proportion of cases detected and the sensitivity at higher quality health facilities which need not be the same as the health facilities where only blood culture is performed. Thus the true blood culture sensitivity or proportion of cases identified at a primary health facility or a remote health facility or a community based health facility where typhoid fever surveillance is conducted may differ. Eight, most studies had small sample size; largest being 103, resulted in wide confidence intervals. Studies with larger sample size in future will help in reducing the uncertainty. Finally, the literature search only included studies published in English which may have resulted in missing some papers published in other languages.

## Conclusion

The proportion of *S*. Typhi detected by blood culture estimated from this systematic literature review provides a point estimate and range for correcting missed typhoid fever cases in disease burden studies based on a scientific rationale. The estimated blood culture sensitivity in this review is higher than the commonly reported value. We question the use of blood culture sensitivity as a proxy for the proportion of *S*. Typhi cases detected in disease burden measures as it underestimates the real problem. This information should be used by clinicians, epidemiologists and decision makers in making rational and logical decisions. Future research should focus on measuring proportion of typhoid fever cases detected by blood culture based on standardized concurrent blood and bone marrow culture or other reasonable reference standard in the field settings which will help in understanding the true proportion of *S*. Typhi cases identified by blood culture.
